# Method to identify and minimize artifacts induced by fluorescent impurities in single-molecule localization microscopy

**DOI:** 10.1117/1.JBO.23.10.106501

**Published:** 2018-10-17

**Authors:** Janel L. Davis, Biqin Dong, Cheng Sun, Hao F. Zhang

**Affiliations:** a Northwestern University, Department of Biomedical Engineering, Evanston, Illinois, United States; b Northwestern University, Department of Mechanical Engineering, Evanston, Illinois, United States

**Keywords:** single-molecule localization microscopy, fluorescence spectral imaging, single-molecule spectroscopy, fluorescent impurities

## Abstract

The existence of fluorescent impurities has been a long-standing obstacle in single-molecule imaging, which results in sample misidentification and higher localization uncertainty. Spectroscopic single-molecule localization microscopy can record the full fluorescent spectrum of every stochastic single-molecule emission event. This capability allows us to quantify the spatial and spectral characteristics of fluorescent impurities introduced by sample preparation steps, based on which we developed a method to effectively separate fluorescent impurities from target molecules.

## 1 Introduction

The term fluorescent impurity usually refers to unintended fluorescence emission from unknown molecules or chemical complexes. The presence of fluorescent impurities represents a long-standing issue in single-molecule imaging and spectroscopy.^1^^–^^3^ To reduce the impact of these fluorescent impurities, stringent cleaning and sample preparation techniques need to be utilized.^1^^–^^3^ In recent years new imaging techniques, such as single-molecule localization microscopy (SMLM),^4^^–^^9^ emerge to offer super-resolution single-molecule imaging far beyond the diffraction limit of the light. However, the impact of fluorescent impurities on correctly interpreting single-molecule imaging results has not been thoroughly investigated.^10^^–^^13^

In conventional fluorescence microscopy, fluorescent impurities are often negligible due to their apparent lower absorption cross-sections and weak fluorescent emissions.^14^^–^^16^ However, growing evidence has shown that fluorescent impurities significantly impact SMLM by inducing imaging artifacts, which include sample misidentification and higher localization uncertainty in cases where fluorescent impurities overlap in space with target molecules.^11^^–^^13^ Although SMLM accumulates the stochastic emissions from individual fluorophores and proteins to collectively render super-resolution images,^4^^–^^6^^,^^8^^,^^9^ the required high-power-density illumination to excite stochastic emissions also unfavorably intensifies emissions from fluorescent impurities.^13^^,^^17^^,^^18^ When a large number of photons are stochastically emitted from fluorescent impurities, they behave similarly to target molecules and are difficult to distinguish and remove.^12^^,^^13^^,^^18^ Preventing sample misidentification is a particularly significant challenge when imaging low number density ($<1\;\mu m^{-2}$) single molecules without distinct structural or morphological features.^10^^,^^11^

Currently, the reported methods to identify target molecules in reconstructed SMLM image mainly rely on spatial and temporal profiling of their stochastic emissions, such as width of the fitted point-spread-function,^19^ repetition rate of blinking events,^20^ and emission intensity.^13^^,^^19^ Emission intensity in particular is commonly compared against a user-defined intensity threshold and one can remove any emission with lower intensity than the threshold, hoping to exclude fluorescent impurities.^11^^,^^13^^,^^19^ However, due to their diverse origins, emissions from fluorescent impurities can often exceed the threshold value, resulting in low specificity.^11^^,^^13^ A more specific criterion is needed to faithfully identify target molecules while rejecting fluorescent impurities. The spectra of all stochastic emissions can be such signatures; however, existing SMLM technologies are unable to measure these spectra. Recently, we and other groups reported spectroscopic single-molecule localization microscope (sSMLM),^17^^,^^18^^,^^21^ which simultaneously detects the spatial and spectral information of each stochastic fluorescent emission event. Hence, we anticipate that sSMLM, by analyzing emission spectrum of every stochastic emission, will provide a highly specific criterion to identify target molecules and to reject fluorescent impurities. In this study, we seek to answer two questions: (1) is it possible to reduce or ultimately eliminate fluorescent impurities and (2) can we utilize the emission spectra to remove fluorescent impurities from all the detected stochastic emissions in a low number density sample.

## 2 Methods and Materials

### 2.1 Coverslip Cleaning

Fisherbrand™ 22×22  mm #1.5 borosilicate coverslips (Fisher Scientific) and precleaned FisherFinest™ 22×22  mm #1 borosilicate coverslips (Fisher Scientific) were imaged using a 532-nm laser at four typical power densities (1.5 to $5.7\;\mathrm{kW}/{\mathrm{cm}}^{2}$) used in SMLM. Before imaging, the coverslips were air blown to remove any large particles. Additional cleaning processes were performed on Fisherbrand™ coverslips as described below.

#### 2.1.1 Piranha solution

A beaker was cleaned and placed in a fume hood. Sulfuric acid ($H_{2}{\mathrm{SO}}_{4}$) (Sigma Aldrich) was added to hydrogen peroxide ($H_{2}O_{2}$) (Sigma Aldrich) at a ratio of 3∶1 (90 to 30 mL).^22^ The coverslips were submerged in the solution for 20 min. The coverslips were then submerged in distilled nuclease-free water (Ambion, ThermoFisher) and then dried by air blowing. The piranha solution was allowed to cool disposal in an appropriate waste container.

#### 2.1.2 Potassium hydroxide and ultraviolet light sterilization

The coverslips were sonicated in 1 M potassium hydroxide (KOH) (Sigma Aldrich) for 15 min.^6^ The coverslips were then rinsed in Milli-Q water and dried using nitrogen ($N_{2}$) gas. The cleaned coverslips were placed in a Petri dish and sterilized using UV light for 30 min.^6^

#### 2.1.3 Hydrochloric acid and prop-2-anol cleaning

Each coverslip was sequentially submerged for 30 s in 36% hydrochloric acid (HCl) (Sigma Aldrich), Milli-Q water, and then prop-2-anol (Sigma Aldrich) before drying with nitrogen ($N_{2}$) gas.^23^

#### 2.1.4 Ultraviolet and ozone cleaning

Coverslips were placed in the ZoneSEM Cleaner^24^ (Hitachi) and exposed to ozone activated by UV light for 2 min per side.

#### 2.1.5 Plasma cleaning

The operating conditions for the plasma cleaner (PC 2000, South Bay Technology) for a mixture of argon and oxygen gas were set to use a forward power of 20 W and a minimized reflection power. A cleaning time of 2 min was selected and a precleaning step was performed to clean the chamber.

The coverslips were placed in glass Petri dishes and plasma cleaned uncovered for 2 min.^25^^,^^26^ Metal tweezers used for handling the coverslips were plasma cleaned during this cycle. Using the cleaned tweezers, the coverslips were turned over and the exposed surface was cleaned using the same settings. Cleaned coverslips were stored in sealed glass Petri dishes.

### 2.2 Coverslip Functionalization

Plasma cleaned coverslips were functionalized via poly-L-lysine (7-octen-1yl) trimethoxysilane (silane) and biotinylated bovine albumin serum (BSA) and neutravidin.

#### 2.2.1 Poly-L-Lysine

Coverslips were incubated in 1 ppm poly-L-lysine^27^ (Sigma Life Science) solution for 20 min. The surface was then rinsed three times using nuclease free water (Ambion, ThermoFisher) before air blowing.

#### 2.2.2 Silanization

A 250 mL Pyrex crystallizing dish was tripled rinsed using methanol (Sigma Aldrich) and then n-heptane (Sigma Aldrich). Working in a chemical hood, 100 mL of n-heptane was added to the dish and 100  μL of (7-octen-1yl) trimethoxysilane^22^^,^^23^ (Sigma Aldrich). Coverslips were added to the silane treatment using tweezers and left overnight in a desiccator without a vacuum. The next day, the coverslips were sequentially sonicated for 5 min in n-heptane, Milli-Q water, and finally chloroform (Sigma Aldrich) before drying using air.

#### 2.2.3 Bovine albumin serum-biotin-neutravidin

Coverslips were rinsed three times with 500  μL phosphate-buffered saline (PBS) (Gibco, Life Technologies). The coverslips were then incubated for 5 min in 200  μL of 0.5  mg/mL biotinylated BSA-biotin^28^ (Sigma Aldrich) in PBS. The BSA-biotin solution was removed, and the coverslip was triple rinsed in 500  μL PBS then incubated for 5 min in 200  μL of 0.5  mg/mL neutravidin^28^ (Invitrogen, ThermoFisher) in PBS. The coverslips were then triple-rinsed in 500  μL immobilization buffer [PBS supplemented with 10 mM of magnesium chloride (${\mathrm{MgCl}}_{2}$) (Ambion, ThermoFisher)]. During imaging, water was used to prevent the treatment from drying. A second surface with glucose-oxidase imaging buffer was also tested.

### 2.3 Immobilization Buffer and Oxygen Scavenger System

Immobilization buffer containing 10 mM ${\mathrm{MgCl}}_{2}$ in PBS (pH 7.4) was freshly prepared and added to the BSA-biotin-neutravidin sample. The immobilization buffer was supplemented with an oxygen scavenging system containing 0.5  mg/mL glucose oxidase (Sigma Aldrich), 40  μg/mL catalase (Sigma Aldrich) and 10% (w/v) glucose (Sigma Aldrich), and 143 mM 2-mercapethanol (Sigma-Aldrich).

### 2.4 Reagent Purity

Purity information for the chemical reagents and proteins used in this study is detailed in Tables 1 and 2, respectively.

**Table 1:** Summary of chemical reagents used in this study.

Chemical	Supplier, product number	Purity (%)	Notes
Ethyl alcohol-200 proof	Sigma Aldrich, 459,844	≥99.5	ACS reagent
2-propanol	Sigma Aldrich, 650,447	99.9	HPLC plus
Potassium hydroxide pellets	Sigma Aldrich, 306,568	99.99	Semiconductor grade
Hydrogen peroxide solution	Sigma Aldrich, 316,989	99.999	Semiconductor grade
Sulfuric acid	Sigma Aldrich, 258,105	95 to 98	ACS reagent
α-D-glucose, anhydrous	Sigma Aldrich, 158,968	96	
2-Mercaptoethanol	Sigma Aldrich, 63,689	≥99.0	BioUltra
Trimethoxy(7-octen-1-yl) silane	Sigma Aldrich, 452,815	80	Technical grade
n-Heptane, anhydrous	Sigma Aldrich, 246,654	99	
Chloroform	Sigma Aldrich, 650,498	≥99.9	HPLC-plus

**Table 2:** Summary of proteins used in this study.

Protein	Supplier, product number	Purity	Notes
Glucose oxidase aspergillus niger	Sigma Aldrich, G2133	≥60% protein	
Poly-L-Lysine	Sigma Aldrich, P4707	Lysine concentration ≥0.45 mmol	Sterile-filtered
Neutravidin, iyophilized powder	Thermo Scientific, 31000	14 μg/mg active protein	Salt free
Albumin, biotin labeled bovine, lyophilized powder	Sigma Aldrich, A8549	80% protein	
Catalase	Sigma Aldrich, C40	≥10,000 units/mg protein	≤0.2 wt. % Thymol

### 2.5 Spectroscopic and Single-Molecule Localization Microscopy Experimental Setup

**Fig. 1:**
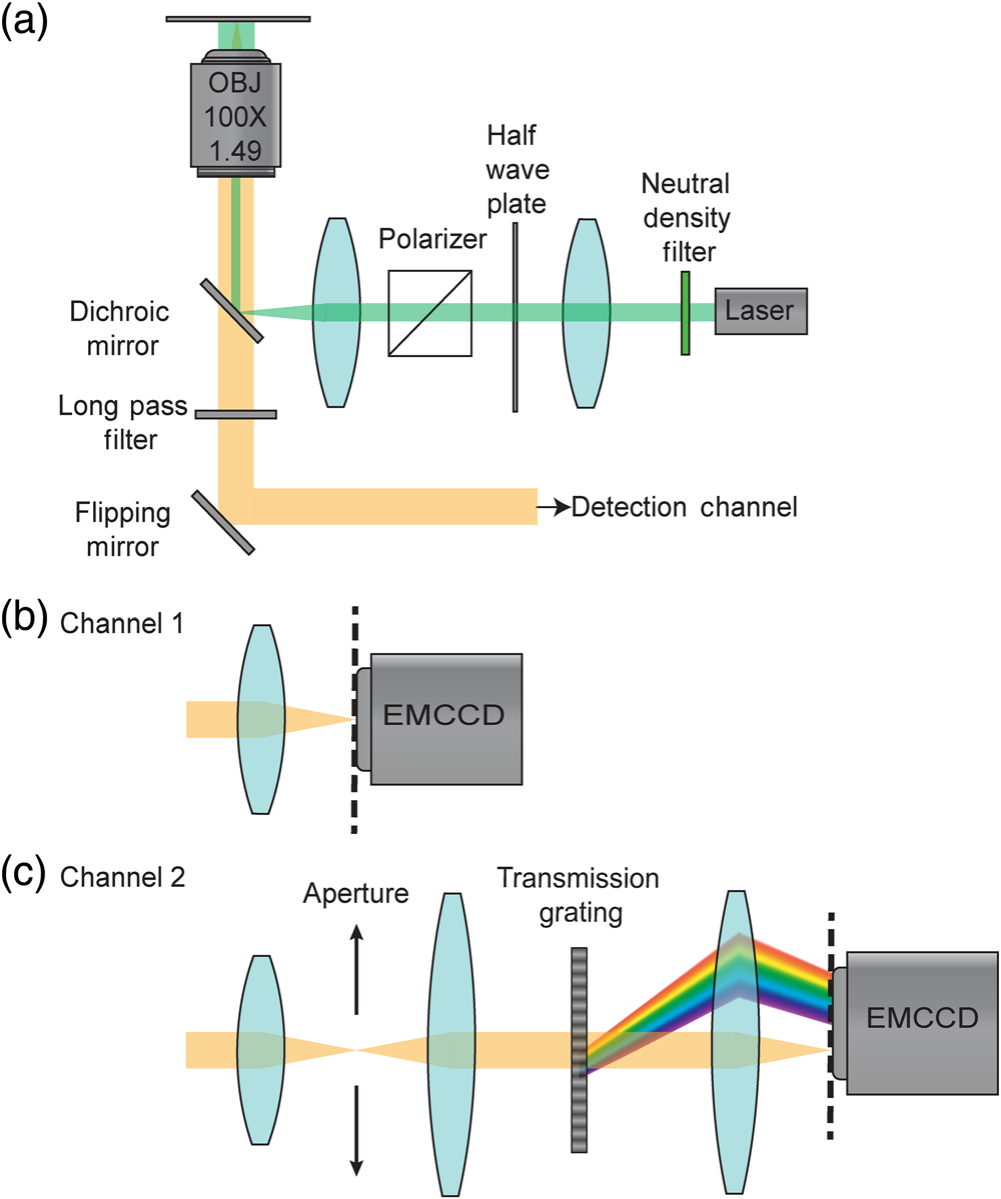
Schematics of SMLM and sSMLM experimental systems. (a) Excitation optics and instrumentation, (b) SMLM detection channel used to capture images of cleaned and functionalized surfaces, and (c) sSMLM detection channel used to capture spatial and spectral images simultaneously.

In these experiments, a diode-pumped solid-state 532-nm laser with a maximum output power of 300 mW was used to illuminate the sample. The laser output was filtered (LL01-532-12.5, Semrock) and passed through a half-wave plate and a linear polarizer to control the output power. The laser was then coupled to an inverted microscope body using a telescopic system and dichroic mirror to focus the light on the back focal plane of a Nikon CFI apochromat total internal reflection objective lens (100×, 1.49 numerical aperture) shown in Fig. 1(a). Adjusting the position of the beam path to the edge of the objective allowed for illumination at the critical angle at the water–coverslip interface, thus limiting the volume of material illuminated. A long-pass filter (BLP01-532R-25, Semrock) was used to reflect the 532-nm laser. SMLM was performed using only position data collected using an EMCCD (iXon 512B, Andor) as shown in Fig. 1(b). For sSMLM, light was guided through a home-made spectrometer equipped with a 100  lines/mm blazed transmission grating (STAR100, Panton Hawskely Education Ltd.), which separated the spatial and spectrally dispersed images. The spatial and the spectral information for each emission event was collected simultaneously on different regions of an EMCCD (ProEm HS 512X3, Princeton Instruments) as shown in Fig. 1(c).

### 2.6 Optical Power Density Measurements

We used a power meter (Newport 1918-R) with a high-power detector (Newport, 918D-SL-OD2R) to measure the power of the excitation laser after beam expansion and before entering the microscope. In comparing with the power measured right after the objective lens, we found a 76% transmission within the microscope body. For all experiments, the power was measured before entering the microscope and scaled by the transmission loss. Power density measurements of 1.5, 3.0, 4.4, and $5.8\;\mathrm{kW}\;{\mathrm{cm}}^{-2}$ at the sample plane were calculated from power measurements at the microscope base (25, 50, 75, and 100 mW) and an illumination radius of 20  μm. The power level was adjusted by changing the angle of the linear polarizer. To calibrate this process, corresponding angles for each power level were recorded and used for all experiments.

### 2.7 Imaging Procedure for Quantitatively Assessing the Origin of Fluorescent Impurities

One coverslip from each treatment was imaged under 532-nm illumination. Five positions on the coverslip were randomly selected and 1000 frames were recorded using an integration time of 10 ms. While imaging cleaned surfaces a 200-×200-pixel field of view (FOV) was used and a 256-×256-pixel FOV was used for imaging functionalized surfaces. For comparison, the number of fluorescent impurities was normalized by the area of their respective FOVs.

To investigate the impact of excitation power density on the number of detectable fluorescent impurities, Fisherbrand™ (Fisher Scientific) and Fisherfinest™ (Fisher Scientific) coverslips were imaged at four different power density levels (1.5 to $5.8\;\mathrm{kW}\;{\mathrm{cm}}^{-2}$). For each dataset, a maximum intensity projection (MIP) image was generated and the number of fluorescent impurities per FOV was determined using the ImageJ plugin ThunderSTORM. There was an average of $2.0\times 10^{7}/{\mathrm{cm}}^{2}$ fluorescent impurities from Fisherbrand and $1.7\times 10^{7}/{\mathrm{cm}}^{2}$ fluorescent impurities from Fisherfinest™ coverslips before cleaning (see Appendix A for more details). As the tested power densities did not have a further impact on the number of fluorescent impurities, we used a typical SMLM power density of $3\;\mathrm{kW}\;{\mathrm{cm}}^{-2}$ in our investigations.

Spectroscopic information from the surfaces was collected by randomly selecting multiple FOVs on a Fisherbrand™ coverslip before cleaning and a plasma cleaned coverslip functionalized with poly-L-Lysine (see Appendix A for additional results). Each FOV was imaged until photobleaching occurred. We captured 1000 frames from the unprocessed coverslip and 3000 frames from the poly-L-Lysine coverslip under 532 nm at $3\;\mathrm{kW}\;{\mathrm{cm}}^{-2}$ with 20-ms integration time per frame.

### 2.8 Spectral Fitting Method

We used a nonlinear least-square fitting method to fit each recorded spectrum to a reference spectrum. As the recorded emission events overlapped in space, the mixed spectrum S attributed to each point spread function can be expressed as follows:

$$S=a_{1}s_{1}(x_{1}+d_{1})+a_{2}s_{2}(x_{2}+d_{2})+w,$$ (1)

where $s_{i}(x)$ is the emission spectrum for each type of molecule at position x, $a_{i}$ is the emission intensity of the molecule, $d_{i}$ is the spectral shift due to conformation heterogeneity of each dye molecule, and w is the error term accounting for additive noise.^14^ Using this equation, parameters for the recorded intensity, spectral heterogeneity, and noise were used to fit experimentally recorded spectra to reference spectra of the dye being studied. The adjusted coefficient of determination ($R^{2}$) was calculated as follows:

$$R_{\mathrm{adj}}^{2}=1-(\frac{n-1}{n-p})\frac{\mathrm{SSE}}{\mathrm{SST}},$$ (2)

where SSE is the sum of the squared residuals {$\mathrm{SSE}=\overset{n}{\underset{i=1}{\sum }}{\left[y_{i}-f(x_{i})\right]}^{2}$}, SST is the total sum of squares [$\mathrm{SST}=\overset{n}{\underset{i=1}{\sum }}{\left(y_{i}-\overset{¯}{y}\right)}^{2}$], n is the number of observations, and p is the number of regression coefficients. The adjusted $R^{2}$ was used to assess the goodness of fitting.

### 2.9 Establishing the Ground Truth within each FOV

We selected 10-nm deoxyribonucleic acid (DNA) origami nanorulers (Gattaquant) labeled with Alexa Fluor 532 and Alexa Fluor 568 to test whether the spectrum could be used to separate target molecules from fluorescent impurities. The nanorulers were the ideal model system for this study as their spacing was unable to be resolved by the 20-nm spatial resolution of SMLM, but their spectral separation was greater than the 3-nm spectral dispersion of our sSMLM. Though the peaks of emission spectra of the dyes used were well separated, both dyes can be directly excited by 532-nm laser. The combined signal from a single resolvable pixel provided a unique spectral signature, which could be used to establish a faithful ground truth for the sample in the presence of fluorescent impurities under low-power density (LPD) excitation of $0.5\;\mathrm{kW}\;{\mathrm{cm}}^{-2}$. We then tested using spectral fitting and intensity thresholding to categorize recorded emission events using high-power density (HPD) excitation of $3\;\mathrm{kW}\;{\mathrm{cm}}^{-2}$.

We observed steady fluorescence emission with rather small temporal fluctuations from all fluorescent point emitters in the LPD condition, we used the average of the 300 frames to extract the spectra with high signal-to-noise ratio. The approximate location of the immobilized nanorulers in the sample was estimated using the average image of each FOV. Overlapping spectra in the average LPD images were removed from the LPD and HPD datasets. Consequently, a total of 15 emitters were excluded from further analysis. Due to their high absorption cross section and quantum yield compared with the fluorescent impurities, we anticipate that the observed fluorescent emissions mainly originated from nanorulers (see Appendix B for more details). The minority of fluorescent impurities excited were removed using the spectral fitting method. The extracted spectra were first normalized using the emission maximum of the record spectra then fit to the reference spectra. We attributed fluctuations in the position of the spectra to conformation heterogeneity of each dye and the influence of noise was ignored in this case. From the reference sample for both dyes, we found that full width half maximum of that emission centroids of Alexa Fluor 532 was 20 nm and Alexa Fluor 568 was 40 nm. We also observed spectral shift parameters of ±10 and ±20 for Alexa Fluor 532 and Alexa Fluor 568, respectively. As 532-nm laser illumination could directly excite 100% of Alexa Fluor 532 and 42% of Alexa Fluor 568, each dye had to exceed the noise floor. Therefore, the background should not exceed 10% of the peak intensities for both dyes. Because Alexa Fluor 532 could be optimally excited using 532-nm laser illumination, the influence of Alexa Fluor 568 was determined by first fitting all 174 points using only the reference spectra of Alexa Fluor 532. The data were then fit using both spectra and the difference in the peak adjusted $R^{2}$ value was used to select a threshold of 0.89 (see Appendix B). Single molecules excited under LPD, which had an adjusted $R^{2}$ value of 0.89 after spectral fitting, were considered to be true nanorulers. The determined spatial and spectral characteristics of the nanorulers established the ground truth for each FOV.

### 2.10 Preparation of Nanoruler Sample

Nanorulers (Gattaquant) DNA origami samples were prepared by adding 1  μL of the nanorulers to 200  μL nuclease free water (Ambion, ThermoFisher). The 10  μL of the nanoruler solution was deposited on a poly-L-lysine coated surface via spin deposition (Laurell WS-650- 23) at 1200 rpm for 30 s.

### 2.11 Imaging Procedure for Nanoruler Samples

**Fig. 2:**
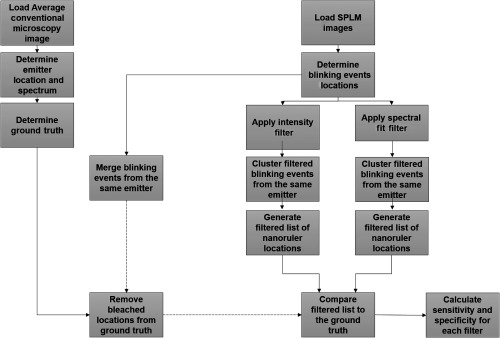
Flowchart of the algorithm used to compare intensity thresholding and spectral fitting filtering methods.

One coverslip containing immobilized nanorulers was imaged under 532-nm illumination. Nine positions on the coverslip were randomly selected and each FOV was imaged using the following procedure. The nanoruler sample was imaged for 4 s (300 frames) at LPD ($0.5\;\mathrm{kW}\;{\mathrm{cm}}^{-2}$). The observed fluorescence from the dye molecules was stable and non-blinking at this power density level. The power density was then increased by changing the polarizer position to reach an HPD ($3\;\mathrm{kW}\;{\mathrm{cm}}^{-2}$) to allow stochastic fluorescence emission of the dye molecules. Images were recorded for 30 s (1500 frames). An integration time of 20 ms was used to record each FOV. These data were used for sample classification as detailed in the algorithm in Fig. 2. The LPD frames were averaged, and the location and spectra were used as references for the single molecule quantification experiments. The HPD frames were used to compare the performance of filters based on emission intensity and spectral fitting.

### 2.12 Sensitivity and Specificity Calculation

We tested the performance of filtering emission events using the emission intensity thresholding and our spectral fitting method. The sensitivity of each method to correctly identify emission events from nanorulers and the specificity of each method to correctly remove emission events from fluorescent impurities were determined by identifying true positives, false positives, true negatives, and false negatives. Nanorulers, which were correctly included by the filtering method, were marked as true positives, while any nanorulers that were excluded were marked as false negatives. True negatives were any fluorescent impurities, which were correctly excluded by the filtering method, while false positives were any fluorescent impurities incorrectly marked as nanorulers. These definitions were used to calculate the sensitivity and specificity of each filtering method using the following equations:

$$\left\{ \begin{array}{c} \text{Sensitivity}=\text{True positives}/(\text{true positives}+\text{false negatives})\\ \text{Specificity}=\text{True negatives}/(\text{true negatives}+\text{false positives}) \end{array}.\right.$$ (3)

### 2.13 Single-Molecule Localization Microscopy Ground Truth

To determine the locations and the number of true nanorulers and fluorescent impurities in each FOV under HPD excitation, incorrect localizations due to background noise were removed from 27,396 recorded points from nine FOVs using a simple density filter. To do this, the nearest neighbors within a 200-nm radius of localization were identified. For clusters with more than five neighbors, the centroid was found and localizations within a 200-nm radius were assigned to that cluster. The average of the localizations was used to estimate the location of the detected emitter. The estimated locations were classified as nanorulers or fluorescent impurities by comparing the results to the ground truth established using the locations and spectra from the averaged image of the same FOV under LPD excitation. On average, we observed 6±2 nanorulers and 35±7 fluorescent impurities among all nine FOVs being measured (see Appendix B for additional details).

### 2.14 Threshold Selection

For both emission intensity thresholding and spectral fitting, the generated histogram from 27,396 emission events was used to select a range of possible thresholds. For intensity thresholding, the background intensity range (120:400) was selected from the histogram of emission intensities to ensure an SNR of at least 6 dB. For spectral fitting, the range (0.8:0.94) was selected from the histogram of adjusted $R^{2}$ values. This range was selected as it fell between two-peak adjusted $R^{2}$ values. Examples using an intensity threshold of 180 and a spectral fitting threshold of 0.84 were compared due to their similar high sensitivities (∼90%).

### 2.15 Filtering Single-Molecule Localization Microscopy Data

For the intensity thresholding method, emission events with an average intensity >180 were classified as fluorescence from nanorulers and all other events were classified as fluorescent impurities. For spectral fitting, the spectrum was first normalized using the maximum intensity of the signal. The accepted spectral shift parameter was ±10  nm for Alexa Fluor 532 and ±20  nm for Alexa Fluor 568. The spectrum from each emission event in the SMLM dataset was fit to the reference and the adjusted R-squared value determined. Emission events with an adjusted $R^{2}$ value >0.84 were classified as fluorescence from nanorulers and all other events were classified as fluorescent impurities.

The localizations identified as emission events from nanorulers were then used to reconstruct SMLM images. For an emitter to be reconstructed, >five emission events within a 200-nm radius of the centroid were required. The location of the emitters after each filtering method was compared with the known location of the nanorulers using the established ground truth. The sensitivity and specificity of each method were then calculated and compared. To estimate the size of each cluster, the standard deviation of emission events within each cluster was used.^29^

### 2.16 DNA Sample Preparation

To further demonstrate our spectral fitting method, we imaged stretched lambda phage DNA (Thermo Scientific, SD0011) labeled with YOYO-1 (Invitrogen, Y3601). The lambda phage DNA was diluted to 100  ng/μL in Tris EDTA (TE) buffer (10 mM Tris and 1 mM EDTA). YOYO-1 dye was diluted to 2  μM in TE buffer. About 32  μL of DNA was mixed with 480  μL of YOYO-1 for a base pair to dye labeling ratio of 5∶1.^25^ The mixture was incubated for 1 h at room temp covered using aluminum foil. The sample was then heated to 65°C for 10 min.^25^ About 50  μL of the labeled DNA was spin stretched on silanized coverslips at 1200 rpm for 30 s.

### 2.17 DNA Sample Imaging and Analysis

We used a 488-nm laser to excite and image the YOYO-1 labeled DNA using sSMLM. About 940 frames of the stretched DNA were captured using at an integration time of 10 ms. The recorded spectrum of each localization was used to calculate the spectral centroid. Color coded sSMLM images were generated using the centroid for each localization. Intensity and adjusted $R^{2}$ values for each localization were used to generate histograms. An intensity threshold of 240 and an adjusted $R^{2}$ threshold of 0.78 were used to remove localizations unrelated to the DNA-YOYO samples. For spectral fitting, the reference spectrum of YOYO-1 was fit to the normalized signal with a spectral shift parameter was ±5  nm. The reference spectrum and selected spectral shift parameter were based on measurements of YOYO-1 bound to DNA immobilized on a glass surface.

## 3 Results and Discussion

**Fig. 3:**
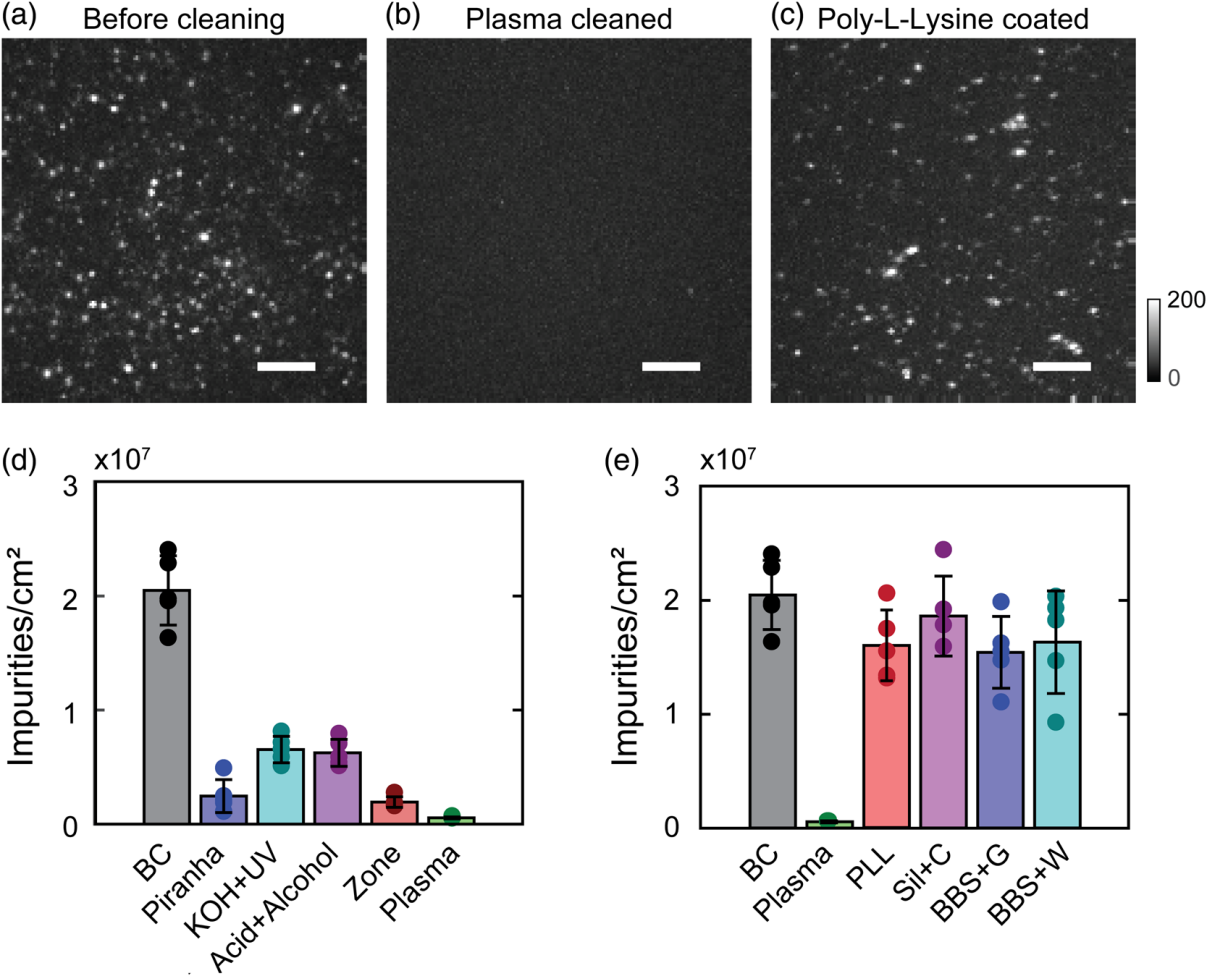
The origins of fluorescent impurities. MIP images (bar: 5  μm) of unlabeled glass surface (a) before cleaning, (b) after plasma cleaning, and (c) after poly-L-lysine functionalization. (d) Comparing densities of fluorescent impurities from five different FOVs before surface cleaning (BC) and after cleaning via the piranha solution (Pir), rinsing with potassium hydroxide and sterilization using UV light (KOH + UV), rinsing with HCl and prop-2-anol (acid + alcohol), exposure to UV-activated ozone (UV-zone) and exposure to argon and oxygen plasma (plasma). (e) Comparing densities of fluorescent impurities for five different FOVs on surfaces before and after plasma cleaning (as a reference) and plasma-cleaned surfaces after functionalization via poly-L-lysine coating, silanization with a final wash of chloroform (Sil + C), bovine-serum albumin and neutravidin (BBS) functionalization with glucose oxidase buffer (BBS + G) and BBS water as the buffer (BBS + W).

To quantitatively understand the origin of fluorescent impurities, we first focused on the essential initial step in sample preparation: preparing optically transparent substrate via various established surface cleaning^6^^,^^22^^–^^25^^,^^30^ and functionalization^22^^,^^23^^,^^28^^,^^31^ methods (see Appendix A for details). We recorded SMLM images of the unlabeled glass substrates (Fisherbrand™, Fisher Scientific) [Figs. 3(a)–3(c)]. As shown in Fig. 3(a), the representative MIP of SMLM images from a nonprocessed glass substrate clearly shows the existence of stochastic fluorescent emission with an average number density of $2.0\pm 0.3\times 10^{7}\;{\mathrm{cm}}^{-2}$ [Fig. 3(d)]. Without adding fluorescence dye, such observed stochastic emission can only be contributed by fluorescent impurities. These observed fluorescent impurities are likely caused by contaminants introduced during the manufacturing, packing, and transportation stages, which may potentially be removed by cleaning the substrate.

Second, we tested literature-reported cleaning methods, including three chemical methods (piranha solution,^22^ KOH solution,^6^ and HCl solution^23^) and two physical methods (UV-ozone^24^ and plasma cleaning^25^^,^^30^). The MIP of SMLM images of the substrate after plasma cleaning is shown in Fig. 3(b) (see Appendix A for results of other cleaning methods). As expected, we found that all tested surface cleaning methods effectively reduced the number of fluorescent impurities [Fig. 3(d)]. Using piranha solution, KOH solution, and HCl solution, the fluorescent impurity number density dropped to $2.5\pm 1.4\times 10^{6}\;{\mathrm{cm}}^{-2}$, $6.4\pm 1.1\times 10^{6}\;{\mathrm{cm}}^{-2}$, and $6.2\pm 1.2\times 10^{6}\;{\mathrm{cm}}^{-2}$, respectively. Using physical cleaning methods, the fluorescent impurity number density, respectively, dropped to $1.7\pm 0.1\times 10^{6}\;{\mathrm{cm}}^{-2}$ and $5.5\pm 0.9\times 10^{5}\;{\mathrm{cm}}^{-2}$ after UV-ozone and plasma cleaning. The fluorescent impurity number density for each cleaning method was calculated using 1000 frames recorded using an integration time of 10 ms and a power density of $3\;\mathrm{kW}\;{\mathrm{cm}}^{-2}$. We hypothesize that while chemical cleaning methods can effectively remove the possible contaminants on the bare substrate, the chemical solution itself may contain new contaminants. Additionally, these methods require rinsing and drying, which could contribute to potential sources of fluorescent impurities. Consequently, these sources of fluorescent impurities reduce the effectiveness of chemical cleaning. Figure 3(d) suggests that plasma cleaning is the most appropriate method in consistently minimizing the occurrence of the fluorescent impurities.

After cleaning, we examined fluorescent impurities introduced by other essential sample preparation steps, which requires a wide variety of chemical reagents and may introduce new sources of fluorescent impurities. To this end, we tested three commonly used surface functionalization methods (poly-L-lysine,^31^ silane,^22^ and biotinylated bovine serum albumin with neutravidin or BBS^28^) after plasma cleaning. We found a significant increase in the fluorescent impurities after the functionalization process [Fig. 3(e)]. Figure 3(c) shows a representative SMLM MIP image after surface functionalization using poly-L-lysine (see Appendix A for results of other functionalization methods). Although we used chemical reagents with the highest purity grade (see Tables 1 and 2 for purity information), we found that the trace amount of fluorescent impurities still imposed significant effects on the fluorescent impurities in SMLM. As shown in Fig. 3(e), after treating with poly-L-lysine, silane solution, and BBS, the observed fluorescent impurities number density increased to $1.6\pm 0.3\times 10^{7}\;{\mathrm{cm}}^{-2}$, $1.9\pm 0.351\times 10^{7}\;{\mathrm{cm}}^{-2}$, and $1.5\pm 0.3\times 10^{7}\;{\mathrm{cm}}^{-2}$, respectively. Adding typical oxygen scavenging imaging buffer (containing glucose, glucose oxidase, catalase, and 2-mercapethanol in PBS supplemented with 10 mM $\mathrm{Mg}{\mathrm{Cl}}_{2}$) to BBS functionalized surfaces further increased the fluorescent impurities number density to $1.6\pm 0.5\times 10^{7}\;{\mathrm{cm}}^{-2}$. Fluorescent impurity number densities were calculated using the same number of frames, integration time, and power density as aforementioned. Clearly, we observed a positive correlation between the fluorescent impurity number density and the use of chemicals, even at the highest available purity grade.^1^^,^^2^ One common practice in single-molecule imaging and spectroscopy is to photobleach the prepared surface prior to sample introduction;^1^ however, any fluorescent impurities associated with the buffer for the sample would be ignored. Additionally, photobleaching could potentially damage or inactivate the functionalized surface if care is not taken to select the appropriate photobleaching power and wavelength.^1^^,^^3^ Therefore, an alternative approach would be necessary to address these problems associated with the removal of all fluorescent impurities. In answering our first question, is it possible to reduce or ultimately eliminate fluorescent impurities, Figs. 3(d) and 3(e) indicate that it is impractical to fully eliminate fluorescent impurities as long as any chemical reagent is used. These results further suggest that researchers should take precaution of the impact of fluorescent impurity in interpreting single-molecule imaging results and underscores the need for a strategy is to distinguish fluorescent impurities in SMLM.

**Fig. 4:**
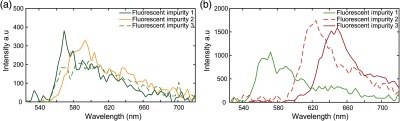
(a) Representative spectra from three fluorescent impurities on a Fisherbrand™ coverslips before cleaning. (b) Representative spectra from three fluorescent impurities associated with poly-L-lysine functionalization.

We hypothesize that sSMLM is more effective to identify target molecules and reject fluorescent impurities. To test this, we first recorded the spectra of fluorescent impurities associated with surfaces before cleaning and after functionalization. Figure 4(a) shows representative spectra of fluorescent impurities in Fig. 3(a). Although fluorescent impurities 1 and 2 have spectra at 569 and 593 nm, respectively, the spectrum of impurity three ranges from 566 to 610 nm. Figure 4(b) shows three representative spectra from fluorescent impurities associated with poly-L-lysine functionalization. We found that these fluorescent impurities displayed a significant amount of inhomogeneity with the different fluorescent impurities having spectra at 562, 623, and 642 nm. These findings indicate that fluorescent impurities have diverse spectral characteristics and can emit a large number of photons when excited using high-power densities. Though the nature of fluorescent impurities remains unknown, their spectral signatures can be used to guide experimental design and data analysis.

**Fig. 5:**
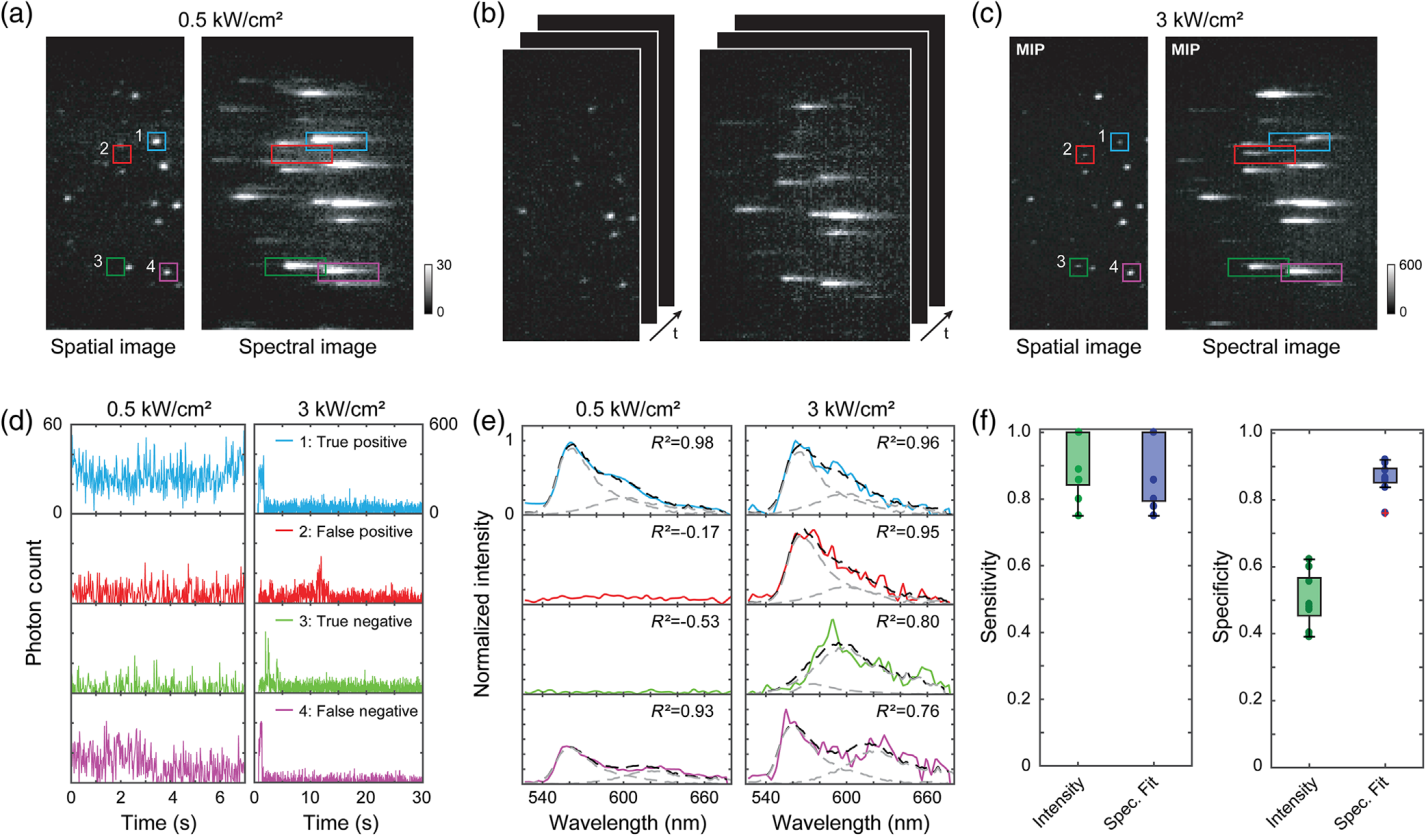
Identifying fluorescent impurities in SMLM. (a) Average spatial and spectral image of DNA origami nanorulers, containing two emitting points labeled with single Alexa Fluor 532 and Alexa Fluor 568 molecules 10 nm apart, immobilized on a poly-L-lysine coated surface. Images were acquired under illuminations with power densities associated with conventional fluorescence imaging ($0.5\;\mathrm{kW}/{\mathrm{cm}}^{2}$). (b) Stack of 1500 frames of the spatial and spectral images of the nanoruler sample for sSMLM ($3\;\mathrm{kW}/{\mathrm{cm}}^{2}$) using the same FOV. (c) MIP images of the spatial and spectral of the same FOV. (d) Photon count versus time from two selected nanorulers (1, 4) and two selected fluorescent impurities (2, 3) highlighted in average and MIP of sSMLM images. (e) Corresponding spectra of the point sources identified in the average and sSMLM images representing true positive, false positive, true negative, and false negative cases for the spectral fitting method. (f) Sensitivity and (g) specificity comparison for nine datasets using an emission intensity threshold of 180 and a spectral fitting filter adjusted $R^{2}$ threshold of 0.84.

Using sSMLM, we developed a spectral fitting method and compared it with the intensity thresholding method to experimentally evaluate their sensitivity in identifying target molecules and specificity in rejecting fluorescent impurities. We used DNA origami nanorulers (labeled with Alexa Fluor 532 and Alexa Fluor 568 with 10-nm spatial separation, Gattaquant)^32^^,^^33^ as the target molecules because the spacing of the dyes was beyond the spatial resolution of SMLM, but their spectral separation was greater than the spectral dispersion of our sSMLM system. We spin-coated the nanorulers on poly-L-lysine functionalized glass substrate. We acquired images within the same FOV using both LPD, $0.5\;\mathrm{kW}\;{\mathrm{cm}}^{-2}$ and HPD, and $3\;\mathrm{kW}\;{\mathrm{cm}}^{-2}$ illuminations. LPD and HPD illuminations, respectively, represented the conditions of conventional fluorescent microscopy and SMLM [Figs. 2(a)–2(c)]. Under LPD illumination, the observed fluorescent emissions are highly likely from the nanorulers [Fig. 5(a)].^34^ Additionally, as photoswitching is suppressed under LPD illumination, the average emission spectrum of the nanorulers and the minority of fluorescent impurities can be recorded. Therefore, to establish the ground truth, we examined and fitted the emission spectra in the average LPD image with known nanoruler emission spectra. Overlapping spectra in the average LPD images were excluded from this analysis. Detected emissions that fit the spectra of Alexa Fluor 532 and Alexa Fluor 568 with an adjusted $R^{2}$ value >0.89 after spectral fitting were considered to be true nanoruler emissions.

We acquired 1500 sSMLM images from the same FOV under HPD illumination [Fig. 5(b)] and plotted both the spatial and spectral MIP images in Fig. 5(c). As the nanorulers have already been identified in the LPD experiment, any additional fluorescent emission identified in HPD experiment can be treated as fluorescent impurities. We compared the sensitivities and specificities of our spectral-fitting method and the commonly used emission intensity thresholding method. We used the histograms for the adjusted $R^{2}$ and emission intensity of each emission event to select a range of possible thresholds. For the spectral fitting method, a range of 0.80 to 0.94 was tested for the adjusted $R^{2}$ values and for the emission intensity thresholding method a range from 120 to 400 was tested allowing the SNR to be at least 6 dB above the background. For fair comparison, we selected the case with ∼90% sensitivities in both methods (Table 2). In this example, for the spectral fitting method emission spectra fitted with an adjusted $R^{2}$ value >0.84 were considered as positive identification of nanorulers, while others were considered as negative identification. On the other hand, in the emission intensity thresholding method stochastic emission with the intensity above 180 will be recognized as a nanoruler, while others were categorized as a fluorescent impurity. We classified nanoruler identifications in the HPD experiments against the ground truth established in the LPD experiments into four categories: true-positive (TP), true-negative (TN), false-positive (FP), and false-negative (FN). Representative intensities and spectra of the four categories are shown in Figs. 5(d) and 5(e), respectively. As shown in Figs. 5(d.2) and 5(d.3), the emission intensity thresholding method would fail to remove both fluorescent impurities as their intensities exceed the established threshold.

We compared the sensitivities [Fig. 5(f)] and specificities [Fig. 5(g)] of both methods using the datasets collected from nine FOVs (see Table 3 for actual values). The sensitivity and specificity for the emission intensity thresholding method are 91%±9% and 50%±8%, respectively; the sensitivity and specificity for our spectral fitting method are 89%±10% and 87%±4%, respectively. Although both methods showed comparable sensitivity in identifying nanorulers, the specificity of rejecting fluorescent impurities by our spectral fitting methods is close to twofold higher than the emission intensity thresholding method. Though an 85% specificity for the emission intensity thresholding method can be achieved by increasing the threshold to 300, this will result in a 13% reduction in sensitivity. On the other hand, the threshold for the spectral fitting method can be increased up to 0.89 allowing for a specificity of 90% with only a 4% reduction in sensitivity. This study shows that the specificity of spectral fitting is less dependent on the user-defined $R^{2}$ threshold than the threshold for emission intensity thresholding. However, due to diverse origins of fluorescent impurities, their spectra can overlap with nanorulers [as shown in Fig. 5(e.2)], which contributed to 13% FP identification in the spectral fitting method. Further reducing FP identification can be accomplished by incorporating additional signatures related to dye photophysics, such as switching time constant^35^^–^^37^ or fluorescence lifetime.^35^^,^^37^

**Table 3:** Sensitivity and specificity comparison between single-molecule filtering based on emission intensity (threshold 180) and spectral fitting (threshold 0.84).

	Intensity threshold	Spectral fitting		Intensity threshold	Spectral fitting
Sensitivity	1.00	1.00	Specificity	0.62	0.91
	0.89	0.78		0.40	0.86
	0.80	0.80		0.47	0.89
	1.00	1.00		0.39	0.76
	0.86	0.86		0.47	0.86
	1.00	1.00		0.48	0.92
	0.86	0.86		0.60	0.87
	1.00	1.00		0.49	0.84
	0.75	0.75		0.56	0.89
Average	0.91	0.89	Average	0.50	0.87
Median	0.89	0.86	Median	0.48	0.87
STD	0.09	0.10	STD	0.08	0.04

**Fig. 6:**
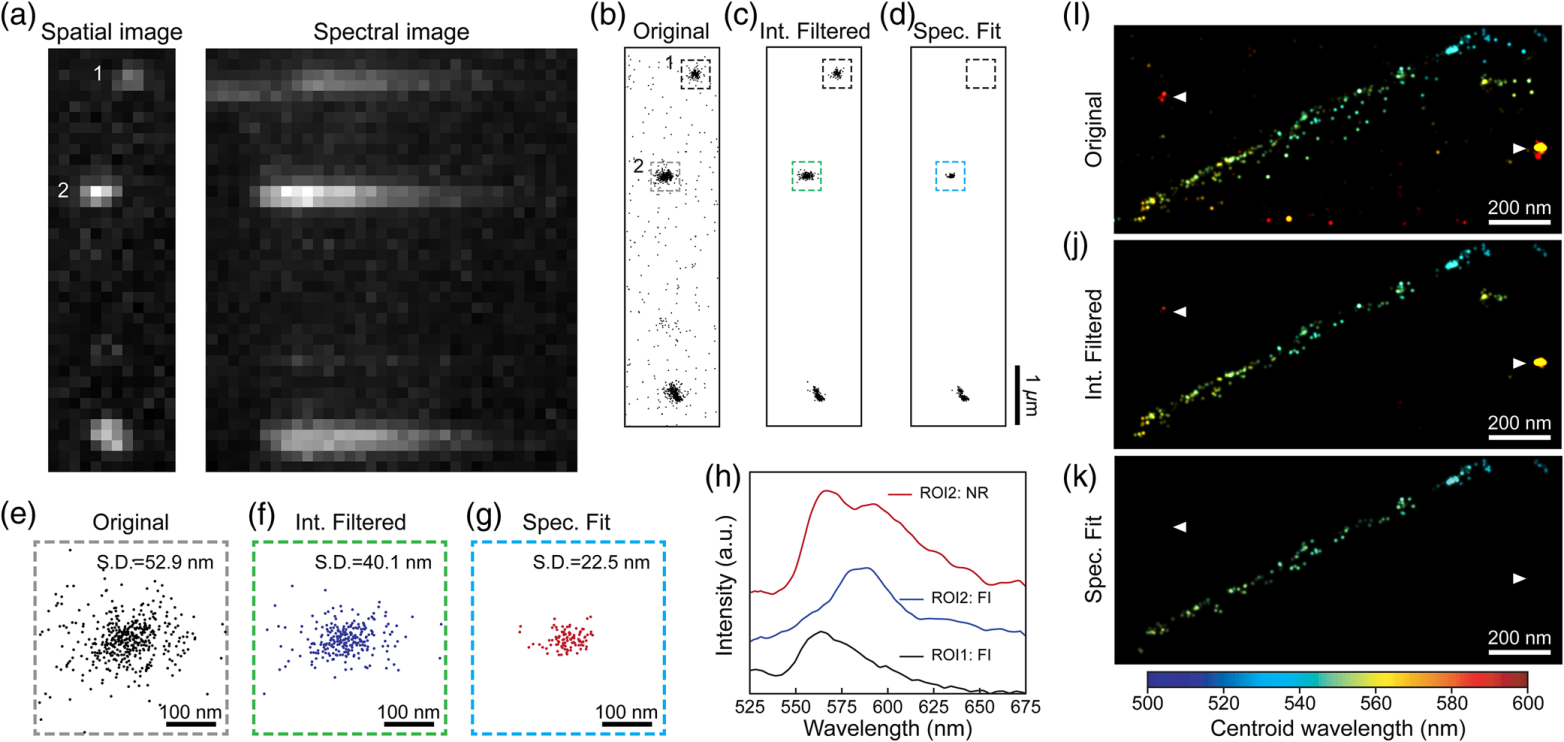
Comparing results in minimizing artifacts induced by fluorescent impurities using intensity filtering and our spectral fitting methods. (a) sSMLM spatial and spectral MIP images of nanorulers with fluorescent impurities. (b) Reconstructed super-resolution images without rejecting fluorescent impurities, (c) result after emission intensity filtering, and (d) result after spectral fitting. ROI1 highlights the localized fluorescent impurities that are eliminated by our spectral fitting method but are misidentified by intensity filtering method. ROI2 highlights the case of spatial overlapping of fluorescent impurities and nanorulers results in higher localization uncertainty. The resulting super-resolution images of ROI2 are further magnified in (e) before filtering [standard deviation (S.D.) 52.9 nm], (f) after intensity filtering (S.D. 40.1 nm), and (g) after spectral fitting (S.D. 22.5 nm). (h) Averaged spectra of fluorescent impurities (FI) and nanoruler (NR) emission. (i) Reconstructed color-coded super-resolution image of stretched lambda phage DNA labeled with YOYO-1 dye on a silane functionalized surface before rejecting emission unrelated to the DNA-YOYO sample (resulting artifacts highlighted by white triangles); (j) result after emission intensity thresholding contains artifacts from unwanted fluorescence; and (k) result after spectral fitting specifically removed artifacts induced by unwanted fluorescence.

Figure 6 shows that our spectral-fitting method better identifies and minimizes artifacts caused by fluorescent impurities. Figure 6(a) shows the sSMLM spatial and spectral MIP images of the same nanoruler sample imaged in Fig. 5, but from a different FOV. We highlighted two regions of interest (ROIs) that contain both nanorulers and fluorescent impurities. Figure 6(b) shows the reconstructed super-resolution image using ImageJ plugin ThunderSTORM^38^ without excluding fluorescent impurities. The results after emission intensity thresholding and spectral fitting are shown in Figs. 6(c) and 6(d), respectively. ROI1 is an example of a misidentified molecule. Within ROI1, among the 189 localized events being originally identified in Fig. 6(b), 114 events were treated by emission intensity thresholding method as nanorulers [Fig. 6(c)]. By comparing corresponding spectra of all the localized events [representative spectrum is shown as the black curve in Fig. 6(h)] with the spectroscopic signature of the nanoruler [Fig. 5(e)], our spectral fitting method determined that none of the 189 events is from nanorulers [Fig. 6(d)]. Using the spectral fitting method in ROI1 prevented sample misidentification. Figures 6(e)–6(g) are the magnified view of the ROI2 shown in Figs. 6(b)–6(d), respectively. ROI2 is an example of a fluorescent impurity that overlaps in space with a nanoruler. Within ROI2, among the 492 localized events being originally identified in Fig. 6(e), which corresponds to a standard deviation (S.D.) of localizations of 52.9 nm.^29^ Among them, 269 events were treated by emission intensity thresholding method as nanorulers, which reduce the S.D. of localizations to 40.1 nm [Fig. 6(f)]. After spectral fitting, we identified 103 events from nanoruler and determined that 389 of the originally identified events were fluorescent impurities. As shown in Fig. 6(h), the representative spectrum of nanoruler (red curve) shows distinct spectroscopic signatures in clear contrast with the spectrum from the fluorescent impurity (blue curve), which further validates the specificity of our spectral fitting method. We demonstrate here that our spectral fitting method can effectively reduce localization uncertainty of samples by removing localizations from fluorescent impurities, with approximately twofold improved localization precision (S.D.: 22.5 nm) comparing with emission intensity thresholding method.

Finally, we compared the performance of emission intensity thresholding and spectral fitting in removing artifacts induced by unwanted fluorescence when imaging DNA samples. For this demonstration, we stretched lambda phage DNA labeled with YOYO-1 on a silane-treated coverslip. We imaged the sample using sSMLM and color-coded the reconstructed image using the spectral centroid for 831 localizations as shown in Fig. 6(i). After applying an intensity filter with an intensity threshold of 240, the reconstructed image contained 476 localizations as shown in Fig 6(j); however, localizations unrelated to the DNA-YOYO sample were not completely removed. We then applied our spectral fitting method with an adjusted $R^{2}$ threshold of 0.78 and found that only 221 localizations were more specifically associated with the DNA-YOYO sample as shown in Fig 6(k). The successful removal of the unwanted SMLM imaging artifacts is highlighted as triangles in Figs. 6(i)–6(k), which results in a clear image after applying our spectra fitting method.

## 4 Conclusion

We show that fluorescent impurities are unavoidable. Although thorough plasma cleaning significantly reduced the number of detectable fluorescent impurities, a large amount of fluorescent impurities can be introduced by required substrate treatments, such as surface functionalization. Although the true origins of fluorescent impurities remain unclear, using sSMLM to perform spectral fitting can effectively improve the specificity of rejecting fluorescent impurities by nearly twofolds comparing with commonly used method while maintaining comparable sensitivity in identifying target molecules. Additionally, we found that the specificity of spectral fitting is less dependent on the user-defined $R^{2}$ threshold than the intensity threshold for intensity filtering. This study suggests that sSMLM, with newly added spectral analysis capability, is a powerful tool for single-molecule studies to guide sample preparation for better experimental design and analysis.

## Figures and Tables

**Fig. 7:**
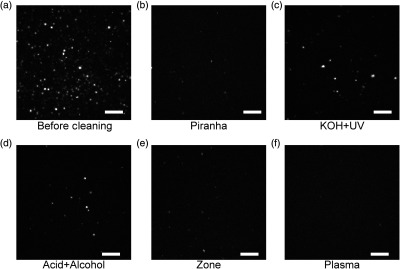
Representative MIP images of a bare Fisherbrand™ coverslip (a) before cleaning, (b) after cleaning using the piranha solution, (c) after sonication in 1 M KOH and sterilization using UV illumination, (d) after rinsing with HCl and prop-2-anol, (e) after cleaning with UV-activated ozone, and (f) after exposure to a mixture of oxygen and argon plasma. All images were captured using 532-nm illumination at a power density of $3\;\mathrm{kW}\;{\mathrm{cm}}^{-2}$. Scale bars are 5  μm.

**Fig. 8:**
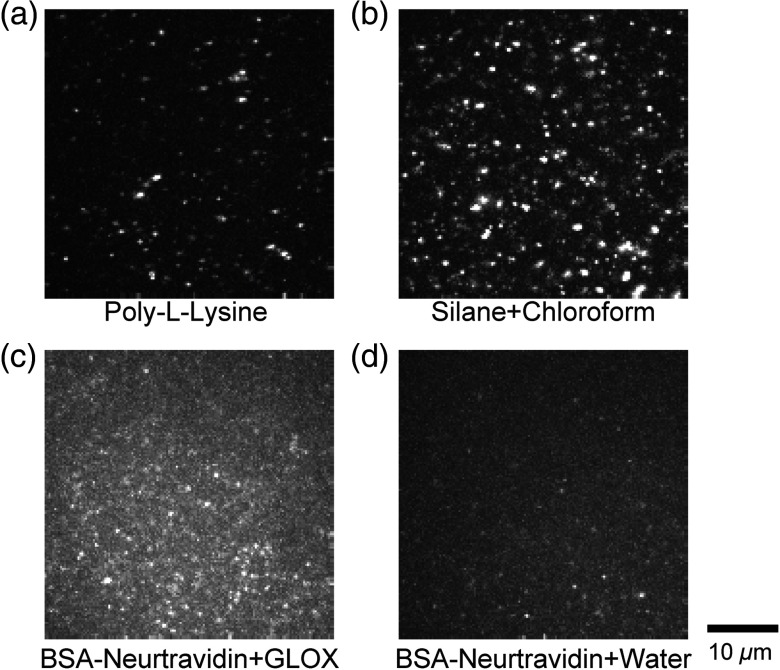
Representative MIP images of plasma-cleaned Fisherbrand™ coverslips functionalized with (a) poly-L-lysine, (b) silane with chloroform as the final rinse, (c) biotinylated BSA and neutravidin with glucose oxidase (GLOX) buffer, and (d) biotinylated BSA and neutravidin with water buffer. All images were captured using 532-nm illumination at a power density of $3\;\mathrm{kW}\;{\mathrm{cm}}^{-2}$.

**Fig. 9.**
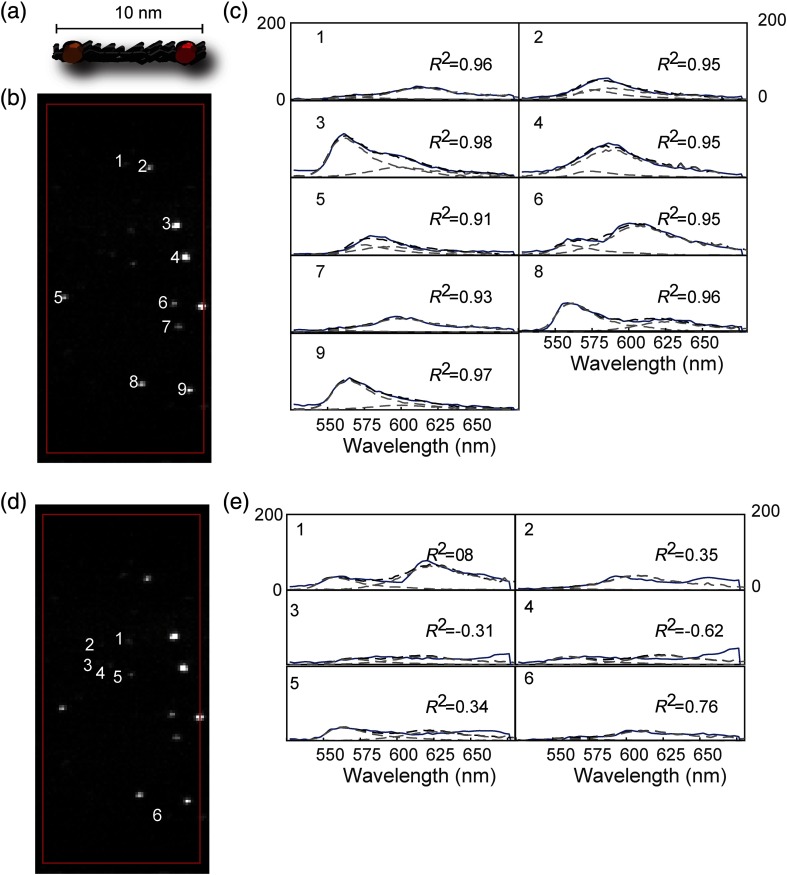
(a) Illustration of the 10-nm Alexa Fluor 532 and Alexa Fluor 568 labeled nanoruler. (b) Representative spatial image, and (c) extracted spectra of nanorulers detected using low power density illumination using the field of view highlighted in Figs. 3(f)–3(j). (d) Representative spatial image and (e) extracted spectra of fluorescent impurities detected using low power density illumination using the field of view highlighted in Figs. 3(f)–3(j).

**Fig. 10.**
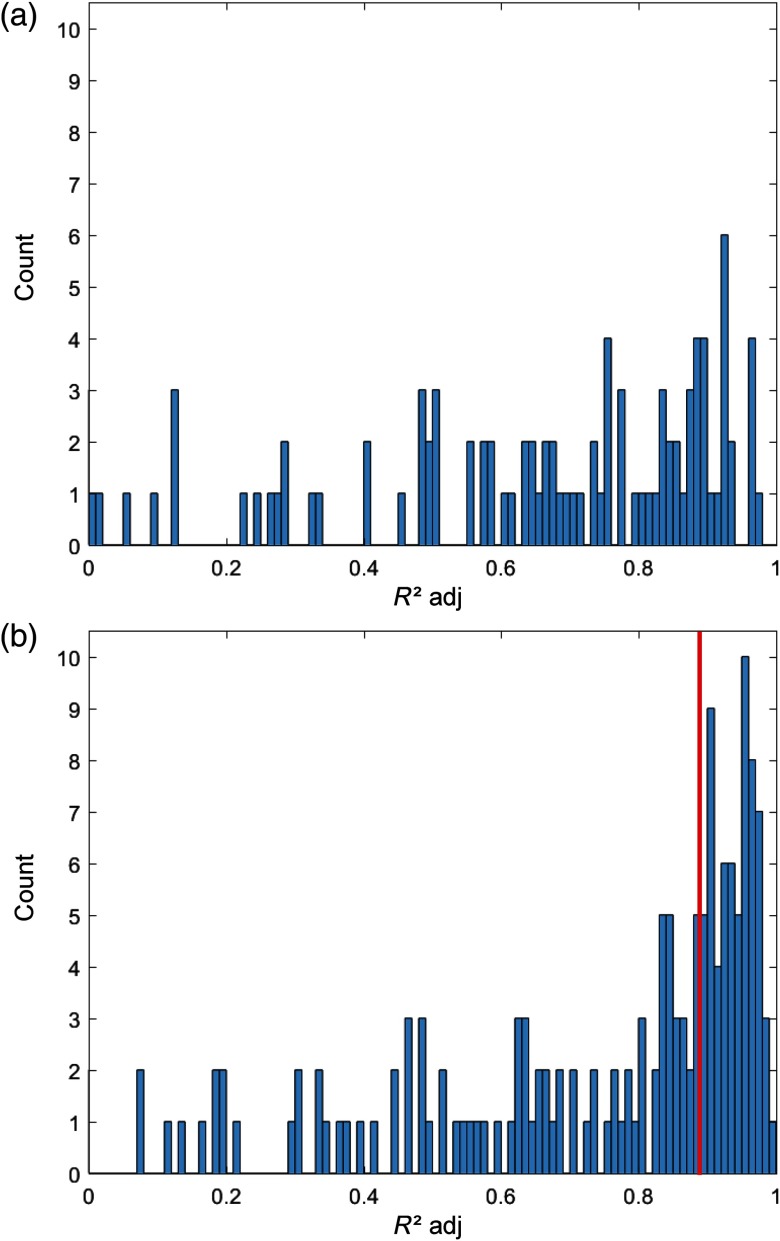
Histograms of adjusted $R^{2}$ values of average spectra from 174 locations in low-power density image after spectral fitting to the reference spectrum of (a) Alexa Fluor 532 and (b) both Alexa Fluor 532 and Alexa Fluor 568 with the adjusted $R^{2}$ threshold of 0.89 highlighted.

**Fig. 11.**
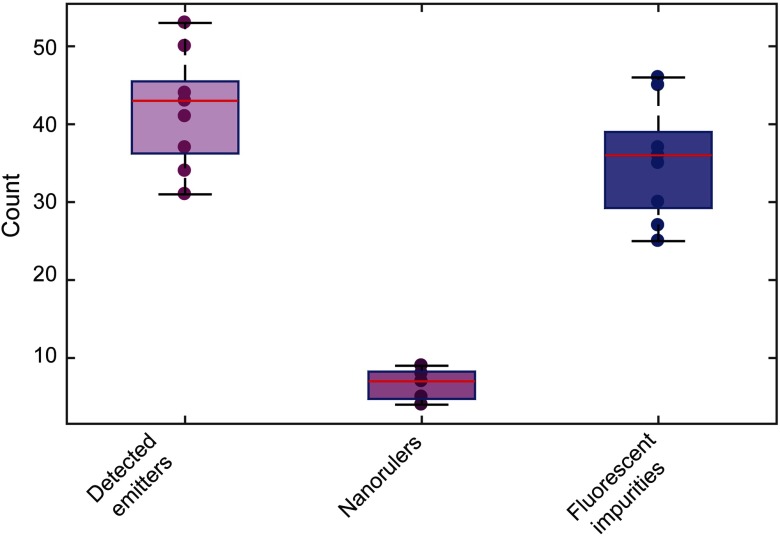
The number of emitters detected using high-power density excitation, the number of nanorulers, and the number of fluorescent impurities detected under high power density excitation for all 9 field of views. The median is highlighted for each box plot.

## Appendix A: Surface Cleaning and Functionalization Results

To identify the origin of fluorescent impurities in SMLM experiments, we first tested their appearance in different steps during sample preparation. As a negative control, bare Fisherbrand™ borosilicate glass before cleaning was imaged using the SMLM setup shown in Figs. 1(a) and 1(b). We found that on average 209±32 fluorescent impurities could be detected using a FOVs of $1.0\times 10^{-5}\;{\mathrm{cm}}^{2}$ at a power density of $3\;\mathrm{kW}\;{\mathrm{cm}}^{-2}$. Higher quality Fisherfinest™ coverslips were also tested and were found to have 173±13 fluorescent impurities within a FOV of $1.0\times 10^{-5}\;{\mathrm{cm}}^{2}$. Five common cleaning methods such as the Piranha solution (Pir), KOH rinsing followed by UV sterilization, hydrochloric (HCl) acid followed by prop-2-anol rinsing, UV-activated ozone (zone) exposure, and plasma exposure were assessed using Fiserbrand™ coverslips. All the cleaning methods significantly reduced the number of fluorescent impurities on the surface with the average number of emitters approximating 25±15 for Pir, 69±12 for KOH, 67±13 for HCl, 18±1 for Zone, and 6±1 for Plasma in same FOVs of $1.0\times 10^{-5}\;{\mathrm{cm}}^{2}$ as shown in Figs. 3(d) and 7. To be noted, chemical methods did not always uniformly clean the surface accounting for the high standard deviation in the number of fluorescent impurities per FOV. This was mostly due to variation in drying the surface. Therefore, care should be given when using chemical cleaning methods as sections of the coverslip may have an accumulation of fluorescent impurities along the direction the coverslip was rinsed. Based on our studies, plasma cleaning was identified as an appropriate method for surface cleaning due to the lowest number of fluorescent impurities and the lowest variability in the number of fluorescent impurities on the surface.

In many experiments, the cleaned surface undergoes additional treatment for proper sample immobilization, optimization of fluorophore performance, and to reduce nonspecific deposition of unwanted molecules.^28^ In this study, three^3^ common surface functionalization methods, poly-L-lysine coating, silanization, and BSA biotin neutravidin functionalization, were investigated using plasma-cleaned surfaces using an FOV of $1.7\times 10^{-5}\;{\mathrm{cm}}^{2}$. All functionalization techniques increased the number of fluorescent impurities on the surface with an average of 277±54 poly-L-lysine, 313±59 for silane, and 259±53 for BSA [see Figs. 3(e) and 8]. We further tested the BSA-biotin neutravidin functionalization using oxygen scavenging imaging buffer and found the average number of fluorescent impurities to be 274±76, respectively, showing that the buffer condition had a minimal impact. However, it was noted that the glucose oxidase buffer increased the overall background of the image by 7%.

## Appendix B: Ground Truth

True nanorulers in this study were identified by analyzing the stable emission spectra of emitters in the LPD datasets ($0.5\;\mathrm{kW}\;{\mathrm{cm}}^{-2}$). Figure 9 shows the spectra of nanorulers and fluorescent impurities. To select a $R^{2}$ threshold for ground truth analysis, the histograms of the $R^{2}$ fitting parameter before and after the influence of the Alexa 568 terms were assessed. As shown in Fig. 10, a threshold of 0.89 was selected to include only emitters whose spectra included both Alexa Fluor 532 and Alexa Fluor 568. Figure 11 shows the number of emitters for all nine FOVs in the HPD datasets ($3\;\mathrm{kW}\;{\mathrm{cm}}^{-2}$) and their categorization as nanorulers or fluorescent impurities after comparison to the established ground truth.
